# Assessment of Motor Performance in Children with Autism Spectrum Disorder: The Relationship Between Clinical Characteristics and Intelligence—An Exploratory Cross-Sectional Study

**DOI:** 10.3390/medicina62010145

**Published:** 2026-01-10

**Authors:** Jenan M. Alhussain, Alaa I. Ibrahim

**Affiliations:** 1 Physiotherapy Unit, Rehabilitation Services Section, Allied Health Department, Jubail General Hospital, Jubail 35713, Saudi Arabia; 2 Department of Physical Therapy, Imam Abdulrahman Bin Faisal University, Dammam 34212, Saudi Arabia; aiibrahim@iau.edu.sa

**Keywords:** autism spectrum disorder, motor performance, CARS scale, intelligence level, Movement Assessment Battery, hand grip, endurance, flexibility

## Abstract

*Background and Objectives*: Evidence on motor performance in children with autism spectrum disorder (ASD) is scarce and inconsistent. The association of motor impairments with autism severity and intelligence remains insufficiently studied. We aimed to examine motor performance parameters in children with ASD compared with typically developing (TD) peers. *Materials and Methods*: In this cross-sectional study, a convenience sample of 26 children with ASD, aged 4–10 years, was recruited from specialized centers in KSA, alongside 27 age- and sex-matched TD children. For the ASD group, severity (Childhood Autism Rating Scale, CARS-2) and intelligence quotient (Stanford–Binet Intelligence Scale, SB5) were extracted from medical records. CARS-2 score was utilized to categorize children with ASD into two groups (mild-to-moderate and severe groups). All study children were assessed for gross and fine motor skills using the Movement Assessment Battery for Children-2 (MABC-2), balance, muscle strength, endurance, and flexibility. *Results*: ASD groups recorded significantly lower scores in all MABC-2 component areas when compared to the TD group (*p* < 0.001). Aiming and catching percentile was significantly lower in the severe ASD group compared to the mild-to-moderate group (*p* = 0.05). Furthermore, children with ASD exhibited increased hypermobility, predominantly at the elbow joints, reduced grip strength, shorter distance in the modified 6 min walk test, and lower standing long-jump performance (*p* < 0.001) when compared to TD group; however, no significant difference was recorded between the ASD groups. Spearman correlation revealed that aiming and catching was negatively correlated with autism severity (CARS-2) (r = −0.38, *p* = 0.05) and positively with IQ (r = 0.51, *p* = 0.03). Aiming and catching was positively correlated with grip strength (r = 0.55, *p* = 0.003), endurance (r = 0.58, *p* = 0.002), and jump distance (r = 0.44, *p* = 0.03), while balance was positively correlated with grip strength (r = 0.44, *p* = 0.02). *Conclusions*: Children with ASD exhibit significant impairments in gross and fine motor performance compared with TD peers, accompanied by hypermobility, reduced strength, and diminished endurance. Notably, aiming and catching ability correlated with both IQ and autism severity as well as specific motor parameters, suggesting its potential as a clinical marker of motor–cognitive interaction in ASD.

## 1. Introduction

Autism spectrum disorder (ASD) is a neurodevelopmental disorder that manifests in early childhood, impacting communication, social interactions, and functional capabilities. The Diagnostic and Statistical Manual of Mental Disorders, Fifth Edition (DSM-5) indicates that previously separate diagnoses, including Asperger’s disorder and pervasive developmental disorder not otherwise specified, are now integrated into a unified diagnostic category of ASD, which illustrates a dimensional spectrum of symptom severity [1]. The main attributes of ASD accommodate problems in interacting and communicating socially, including deficits in verbal and nonverbal communication, inability to use proper body language and eye contact, and ineffective use of gestures. The secondary features are restricted and repetitive patterns of activities that vary between motor stereotypes, echolalia, and excessive or limited interest in specific sensory inputs in the environment [1]. Other physical problems associated with ASD may include sensory impairments, sensory–motor problems, fine and gross motor deficits, muscle weakness, hypotonia, and balance problems [2].

Motor problems among children with autism spectrum disorder have been known of since Kanner [3], an Austrian-American psychiatrist, first described the disorder. He mentioned that “children were essentially normal, have relatively large heads, several of them were clumsy in gait and gross motor performance, but were very skillful in terms of finer muscle coordination” [3]. Nowadays, there is an increasing amount of evidence that sheds light on the motor problems of children with autism spectrum disorder and their need for physical therapy [4].

It is vital to investigate motor impairments in children diagnosed with autism spectrum disorder. First, it may help in understanding the neurophysiological abnormalities associated with ASD. Piochon [5] studied a mouse model of 15q11-13 duplication, which is found in 1–3% of children diagnosed with ASD [6], to explore associated synaptic and neural circuit abnormalities related to ASD. He found that investigating motor behavior provided an advantage over studying social behavior in mice, because there is a vast difference between the social repertoire of mice and that of humans. In contrast, some cerebellar-dependent motor learning skills that involve eye movement are easier to correlate because they are less complex and conserved throughout mammalian evolution.

Second, the motor problems of children with autism spectrum disorder can affect them on a social basis. Stins and Emck [7] mentioned the tremendous impact of this problem, as it leads to reduced participation in play with other children, which, in turn, can aggravate social problems. Lang [8] found that, due to motor learning deficits, individuals with ASD were found to be less engaged in physical activities and exercise and could be deprived of their social, behavioral, physical, and psychological benefits.

Third, early identification of motor abnormalities in children with a confirmed diagnosis of autism spectrum disorder is of great importance in targeting and developing proper interventions, which may help in developing assessment tools that can capture variabilities in motor skills [9]. However, motor assessment should be performed in conjunction with psychometric measures. Yu [10] encouraged considering IQ when assessing fine motor skills in preschool children. He studied the effect of the discrepancy between verbal IQ (VIQ) and performance IQ (PIQ) on motor performance. He found that when verbal IQ (VIQ) score was higher than performance IQ (PIQ) score, there was lower motor coordination and visual–motor integration. Eggleston et al. [11] found that general execution of gross and fine motor activities was highly linked with intellectual ability, but not the degree of autism severity, while praxis error and time to completion were strongly correlated with the degree of autism severity but not intelligence quotient score. Bremer and Cairney [12] suggested an interrelationship between motor impairment and adaptive behavior. However, they suggested that more studies are needed to confirm this correlation.

The current exploratory study sought to achieve the following: (1) Assess motor performance, muscle strength, endurance, and joint mobility in children with ASD compared to their typically developing counterparts. Motor performance parameters included gross and fine motor skills, balance, muscle strength, endurance, and musculoskeletal flexibility. (2) Investigate the correlations between motor performance, autism severity, intellectual abilities, demographic data, anthropometric measurements, and clinical characteristics of those children with ASD.

## 2. Materials and Methods

### 2.1. Study Design and Participants

This cross-sectional, descriptive-analytic study recruited 55 children with ASD from four rehabilitation and day-care centers in the Eastern Province of Saudi Arabia. After applying eligibility criteria and accounting for dropouts, 26 children aged 4–10 years completed the study. A comparison group of 27 typically developing children, matched by age and sex, was selected from local elementary schools. Inclusion criteria required full-term birth, a clinical diagnosis of ASD, and age between 4 and 10 years. Exclusion criteria included neurological disorders (such as tumors, traumatic brain injury, Fragile X syndrome, or cerebral palsy) and medical conditions affecting sensory function, including visual or auditory impairments.

### 2.2. Sample Size

The sample size of the participants was calculated by sample size calculator software (G*Power 3.1.9.2). The calculation was based on the study conducted by Tyler et al. [13] in which hand grip strength was recorded for school-aged children with ASD and their typically developing peers. From this trial, the following values were utilized: mu1 = 17.5, mu2 = 21.1, and sigma (common value of SD) = 9.9. Then, the alpha level and the desired power values were set at 0.05 and 0.80, respectively. The calculated sample size obtained from this trial was equal to a total of 56 children (28 children with ASD and 28 typically developing children), including 20% compensation for any drop out. Sample size was calculated for hand grip strength because we had no available effect size data on the MABC-2 outcomes in children with ASD. Therefore, values should be interpreted as exploratory, and future studies desiring to use standardized motor assessments (e.g., MABC-2) for sample size calculations are recommended to do so.

### 2.3. Clinical Characteristics (Autism Severity and Intelligence)

Information on autism severity and cognitive ability was obtained from the medical records of children with ASD. The severity of autism in this study was evaluated using the Childhood Autism Rating Scale—Second Edition (CARS-2), which was obtained from medical records and administered by certified psychotherapists. While CARS-2 is a validated (Cronbach’s alpha of approximately 0.90 (95% CI, 0.87–0.92)) [14] and commonly utilized tool for measuring autism symptom severity that aligns well with DSM-5 criteria, it does not serve as an observational gold-standard instrument like the Autism Diagnostic Observation Schedule—Second Edition (ADOS-2). As a result, the characterization of severity in this study does not permit detailed domain-specific profiling of social-communication or restricted and repetitive behaviors. Therefore, findings regarding autism severity should be interpreted with due caution. CARS-2 includes 15 items rated on a 4-point scale and categorizes children into showing minimal-to-no symptoms (15–29.5 for ages less than 13 years; 15–27.5 for ages more than 13 years), mild-to-moderate symptoms (30–36.5 for ages less than 13 years; 28–34.5 for ages more than 13 years), or severe symptoms (37 and higher for ages less than 13 years; 35 and higher for ages more than 13 years) [15]. It is considered a strong measure of autism severity, with good sensitivity, specificity, and alignment with DSM-5 criteria [16]. In this study, children were classified as having mild-to-moderate or severe ASD based on their CARS-2 total score.

Cognitive ability was measured with the Stanford–Binet Intelligence Scale, Fifth Edition (SB5), which evaluates five cognitive domains using ten subtests to generate various composite scores, including verbal IQ, nonverbal IQ, and full-scale IQ [17]. The SB5 demonstrates high reliability across its major index scores [16]. For this study, only the final IQ score was used to estimate overall cognitive ability, and because most participants were nonverbal, the performance (nonverbal) IQ score was applied.

In order to minimize confounding variables, children diagnosed with ASD and verified comorbid neurodevelopmental, neurological, or psychiatric disorders (such as ADHD, epilepsy, or cerebral palsy) and individuals who were on medications that influence motor or cognitive abilities (including psychostimulants, antipsychotics, or anticonvulsants), as well as those who had engaged in organized motor-based therapies (like physiotherapy or occupational therapy) in the six months prior, were excluded.

### 2.4. Procedures and Outcome Measures

All study participants (ASD and typically developing children) were examined for their anthropometric characteristics including weight, height, and BMI and their motor performance aspects including gross and fine motor skills, static and dynamic balance, muscle strength, endurance, and musculoskeletal flexibility. Body weight was assessed utilizing a calibrated digital scale, while height was determined with a wall-mounted stadiometer, with participants being barefoot and dressed in light clothing. Body mass index (BMI) was computed by dividing weight (kg) by the square of height (m^2^). For all the following tests, the children were allowed to be familiarized with the test, and then suitable instructions and reinforcement methods were provided as necessary to ensure optimal performance of the child.

#### 2.4.1. Movement Assessment Battery for Children-2 (MABC-2) (Primary Outcome Measure)

**Figure 1:**
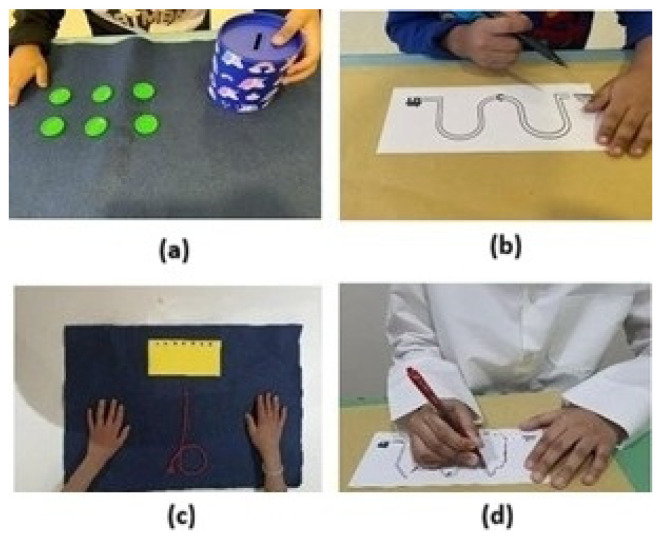
Some component areas of manual dexterity in MABC-2 for age bands 1 and 2. (**a**): Posting coins; (**b**): drawing trail for age band 1; (**c**): threading lace; (**d**): drawing trail for age band 2. Source: Authors’ own work.

MABC-2 is a widely used tool for assessing fine and gross motor skills, including static and dynamic balance, in both typically developing children and those with conditions such as ASD [18]. It is suitable for ages 3–16 years and includes eight tasks per age band across three domains: manual dexterity, aiming and catching, and balance (Figure 1). These produce sub-scores and a total motor score, which are converted to raw, standard, and percentile scores. Raw scores of 0–9.5 indicate normal performance, 10–13.5 indicate borderline performance, and scores above 13.5 suggest motor impairment. Percentile scores are displayed using a traffic-light system: <5% (red) indicates impairment, 5–15% (amber) suggests risk, and >15% (green) indicates no impairment [19]. Research shows the MABC-2 has good clinical value in detecting motor difficulties, especially in Developmental Coordination Disorders, with strong construct validity, good discriminative validity, and acceptable test–retest reliability [19]. Cronbach’s alpha ranged from 0.65 to 0.79, indicating acceptable internal consistency [20]. Because normative percentile data for the MABC-2 are mainly sourced from Western populations, it is essential to interpret percentile-based classifications in Middle Eastern children with caution. In this study, the MABC-2 was predominantly utilized to facilitate standardized comparisons both within the sample and between groups, rather than to make definitive normative conclusions.

#### 2.4.2. Passive Range of Motion

**Figure 2:**
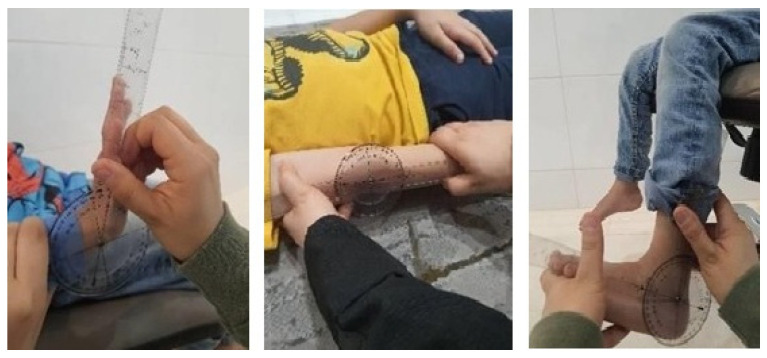
Passive range of motion assessment for wrist, elbow, and ankle joints. Source: Authors’ own work.

Joint flexibility was assessed following Shetreat-Klein’s method [21] by measuring passive range of motion (ROM) in the fingers, wrist, elbow, and ankle using a goniometer (Figure 2). For the fingers, ROM was measured at the metacarpophalangeal joint with the wrist in neutral. Wrist ROM was assessed with the forearm supported and the hand extending beyond the table. Elbow measurements were taken with the forearm supinated. Ankle ROM was evaluated with the knee flexed and the foot held in neutral alignment. In each joint, maximal extension and flexion were performed, and the corresponding angles were recorded.

#### 2.4.3. Muscle Strength

**Figure 3:**
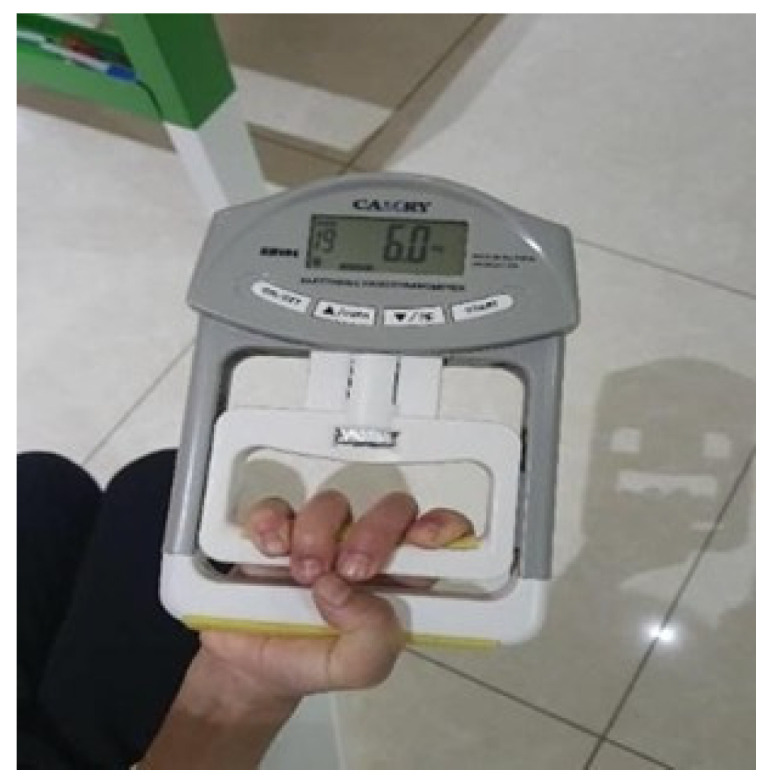
Assessment of grip strength using the dynamometer. Source: Authors’ own work.

Hand grip strength was measured using a calibrated electronic hand dynamometer (Figure 3) (Camry^®^ EH101, Camry Electronic Co., Zhongshan, China). Isometric hand grip strength is recognized as a significant indicator of overall muscle strength [22]. The device is reliable and valid for typically developing children and has also shown strong reliability in studies involving children with ASD [12,23]. Measurements were taken with participants seated comfortably, the arm positioned neutrally, and the elbow extended. After practicing with the device, each child performed a maximal 5 s grip effort with verbal encouragement. Three trials were conducted, with one-minute rests between them, and the highest value was recorded. The device was calibrated before each use, and grip strength was reported in kilograms.

#### 2.4.4. Modified 6-Minute Walk Test (M6MWT)

The modified 6 min walk test (M6MWT) was used to assess endurance by measuring how far each participant could walk in six minutes. This test is valid and reliable in typically developing children [24], and previous research in children with ASD shows acceptable reliability, although results should be interpreted cautiously due to wide confidence intervals [24]. Because children with ASD may have difficulty with attention, motivation, and distraction in open spaces, the test was adapted for this study by using a shorter 15 m indoor walkway instead of the standard 30 m distance. The adjusted 15 m walkway was employed to improve feasibility and adherence in children diagnosed with ASD; consequently, the findings should be understood in a comparative context within the study sample instead of being measured against normative reference values. Participants practiced beforehand, were instructed to walk at a self-paced speed without running and received verbal encouragement at each turnaround point. The total distance walked in six minutes was recorded, followed by a six-minute rest period.

#### 2.4.5. Standing Long Jump (SLJ)

Anaerobic fitness was measured using the standing long jump (SLJ), a valid and reliable test for children with ASD [12]. Participants first practiced up to five maximal jumps with an equal rest period, then performed the test by jumping forward as far as possible with verbal encouragement. The distance from take-off to landing was measured in centimeters, and the average of three recorded trials was used for analysis.

#### 2.4.6. Musculoskeletal Flexibility: Passive Knee Extension Test

**Figure 4:**
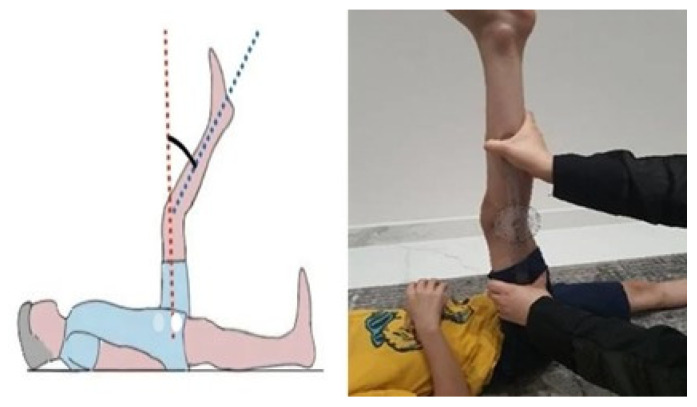
Passive knee extension test. Source: Authors’ own work.

Hamstring flexibility was assessed using a passive knee extension test (Figure 4). Following established procedures [25], the child lay on a firm surface with the tested hip flexed to 120°, while the opposite hip was stabilized with a belt. The knee was then passively extended with a standardized force, and the angle was measured using a goniometer to determine hamstring tightness. This method has shown good inter-tester reliability in adults, although its use has not been specifically validated in children with ASD.

### 2.5. Statistical Analysis

Data analysis was performed using SPSS (version 23). Continuous variables, including age, anthropometrics, muscle strength, range of motion, endurance, and flexibility, were checked for normality using the Shapiro–Wilk test and visual inspection, showing that they were not normally distributed. Means and standard deviations were used for continuous data, while frequencies and percentages described categorical data. Because the data were non-normal, the Kruskal–Wallis test was applied to compare the three groups (two ASD groups and the typically developing group). The Mann–Whitney U test was used to compare the two ASD groups on CARS-2 and IQ scores. Spearman’s bivariate correlations were conducted to identify relationships between motor performance measures, demographics, anthropometrics, autism severity, and intellectual variables.

## 3. Results

### 3.1. Demographic, Anthropometric, and Clinical Characteristics

Table 1 shows the demographic, anthropometric, and clinical characteristics of all study children. Although there was no significant difference in weight and height between the TD and ASD groups, the BMI was significantly higher in the ASD groups compared to the TD children with *p* ≤ 0.05. Handedness did not differ among the three groups. The TD group had an average CARS score (15–29.5), which indicates minimal to no symptoms of ASD, while individuals with mild to moderate ASD reported CARS-2 scores with mean of 31.2 ± 3.6, and those with severe ASD reported a 38.9 ± 2.6 mean CARS-2 score. Individuals with mild to moderate ASD had an average IQ (64.9 ± 25.5), while individuals with severe ASD had an average IQ of 57.7 ± 16.1. The Mann–Whitney test was used to compare the ASD groups with respect to CARS-2 and IQ scores. Although there was a significant difference found in CARS-2 score among the two ASD groups (*p* < 0.001), there was no significant difference in IQ when comparing the ASD groups (*p* = 0.4).

### 3.2. Movement Assessment Battery for Children-2 (MABC-2)

Table 2 illustrates that typically developing (TD) children achieved significantly higher scores than both the mild–moderate and severe ASD groups across all domains of the Movement Assessment Battery for Children-2, which include manual dexterity, aiming and catching, and balance, in terms of both standard scores and percentiles. Kruskal–Wallis tests revealed substantial and statistically significant differences between TD and each ASD subgroup (*p* < 0.001). Conversely, comparisons between MM-ASD and S-ASD did not show any significant differences in manual dexterity or balance (*p* > 0.05), although aiming and catching exhibited a borderline difference solely for the percentile score (*p* = 0.05). The findings highlight a pronounced reduction in gross and fine motor performance among children with ASD when compared to their typically developing counterparts, with relatively comparable motor profiles observed between the mild–moderate and severe ASD groups.

### 3.3. Passive Range of Motion

Table 3 illustrates that children with ASD exhibited diminished passive mobility in several specific joints when compared to their typically developing counterparts. For instance, elbow flexion was found to be lower in both ASD groups (MM-ASD: 135.7°; S-ASD: 133.6°) in contrast to typically developing children (145.6°), and wrist extension was similarly diminished in the ASD groups (approximately 87–88°) compared to the typically developing group (97.7°). Ankle dorsiflexion also presented differences, with the mild–moderate ASD group displaying a significantly smaller range (15.4°) relative to typically developing children (24.9°). Elbow joint hypermobility was more frequently observed in children with ASD (MM-ASD: 5.5°; S-ASD: 4.1°) compared to typically developing peers (2.0°), whereas no consistent hypermobility was identified in other assessed joints. Conversely, the majority of other measurements, such as wrist flexion, finger flexion, finger extension, and ankle plantarflexion, did not reveal any significant differences among the groups, with *p* values < 0.05.

### 3.4. Muscle Strength, Endurance, and Flexibility

Table 4 indicates significantly reduced muscle strength and endurance in children with ASD when compared to their typically developing counterparts. Hand grip strength was notably diminished in both ASD groups (for instance, average grip: TD, 11.1 kg; MM-ASD, 4.2 kg; S-ASD, 3.5 kg; *p* < 0.001), and similar trends were noted for both the right and left hands. Endurance assessments also revealed substantial disparities, with typically developing children covering longer distances in the 6 min walk test (500.6 m) compared to the MM-ASD (353.3 m) and S-ASD (309.4 m) groups, as well as achieving greater standing long-jump distances (136.3 cm versus 21.8 cm and 11.9 cm, respectively; *p* < 0.001). Conversely, musculoskeletal flexibility exhibited fewer differences among the groups; while knee angle measurements were greater in the MM-ASD group (for example, right knee: 12.2° compared to 2.8° for TD; *p* = 0.04), most comparisons of flexibility, including those between MM-ASD and S-ASD, did not reach statistical significance.

### 3.5. Association Between Motor Performance Parameters, Clinical, and Intellectual Characteristics

The correlation analysis in Table 5 reveals that motor performance on the MABC-2 was significantly linked to various clinical and physical performance metrics in children with autism. Increased severity of autism (CARS-2) was moderately correlated with reduced aiming and catching standard score (r = −0.38, *p* = 0.05), whereas IQ exhibited a positive correlation with aiming and catching standard score (r = 0.51, *p* = 0.03). Among the motor domains, aiming and catching displayed the strongest and most consistent correlations with physical performance, significantly correlating with right- and left-hand grip strength (r = 0.59 and r = 0.48, respectively), average grip (r = 0.55), 6 min walking distance (r = 0.58), and standing long-jump distance (r = 0.44). Additionally, balance scores were positively correlated with hand grip strength (r = 0.36–0.49) and average grip (r = 0.44), while total standard scores showed significant positive correlations with grip strength (r = 0.39–0.44) and walking distance (r = 0.42). In summary, enhanced muscle strength and improved endurance were associated with higher motor proficiency on the MABC-2 among children with autism.

## 4. Discussion

The present study aimed to examine differences in motor performance between children with ASD, across varying IQ levels and CARS severities, and their typically developing peers, while also exploring how gross and fine motor skills measured by the MABC-2 relate to specific motor performance parameters. Overall, the findings reveal that children with ASD experience marked motor impairments across multiple domains, with significant associations between select motor tasks and measures of strength and endurance. These results reinforce the growing body of evidence indicating that motor deficits represent a core and pervasive feature of ASD rather than an isolated comorbidity.

One of the initial findings was that BMI was significantly higher in both ASD groups than in typically developing children (*p* ≤ 0.05). This aligns with earlier research demonstrating reduced physical activity levels in ASD [13]. As highlighted by Gülgösteren [26], the COVID-19 lockdown intensified this issue, contributing to weight gain and decreased activity due to restricted outdoor play, disrupted routines, and increased screen time. Reduced participation in physical activities may reflect difficulties with fundamental movement skills (FMSs), which serve as the foundation for more complex motor behaviors [26]. FMSs including locomotor, object-control, and stability tasks are typically acquired and refined throughout childhood. Deficiencies in these areas in ASD may limit engagement in sports, peer play, and structured exercise, thereby contributing to poorer physical fitness and elevated BMI.

Although the two ASD subgroups differed in symptom severity, they did not differ significantly in IQ scores, which ranged from average to severe intellectual disability. Despite this similarity in cognitive level, both ASD groups displayed pronounced deficits on all components of the MABC-2 compared with their typically developing peers, with highly significant differences across manual dexterity, aiming and catching, and balance domains (*p* < 0.001). This supports extensive prior evidence indicating that children with ASD exhibit widespread motor impairments, regardless of age or intellectual functioning [25,26]. Specifically, Craig [27] reported that children with ASD and intellectual disability scored substantially lower on MABC-2 total scores and on all component subtests compared with typically developing children. The present study echoes these findings, as both ASD groups demonstrated significantly poorer performance in all MABC-2 domains.

The skills of aiming and catching were identified as the only domain of the MABC-2 that showed a significant correlation with both the severity of autism and intellectual functioning. Nevertheless, since autism severity was evaluated using the CARS-2 tool instead of a recognized observational standard like ADOS-2, these correlations must be viewed as preliminary. The lack of ADOS-2 restricts the accuracy of symptom profiling and hampers the capacity to link variations in motor performance to particular autism domains. Therefore, performance in aiming and catching should not be regarded as a conclusive indicator of autism severity, but instead as a motor activity that seems to reflect broader neurodevelopmental functioning. Craig [27] similarly found that aiming and catching was disproportionately impaired in children with ASD and intellectual disability. This task requires the integration of visuospatial processing, predictive timing, attention, understanding of others’ intentions, and all processes that may be altered in ASD. The theoretical explanation offered by Craig [27] and others suggests that abnormalities in cerebellar structure and function, along with atypical development of sensorimotor and posterior temporal regions, may contribute to these specific impairments. Children with lower IQ may also require more time to acquire complex motor skills and may have fewer opportunities for exploratory play and skill refinement [27,28]. Consequently, aiming and catching appears to be particularly sensitive to both neurological and cognitive factors in ASD.

In addition to fine and gross motor difficulties, timed elements of the MABC-2 such as manual dexterity tasks and activities on the balance board were notably impaired among ASD participants. Green [29] previously observed that children with ASD struggle with time-dependent tasks, though the underlying mechanism remains unclear. One explanation proposed in the literature involves impaired perception–action coupling [30], in which sensory information is not efficiently integrated to guide movement. Another interpretation is grounded in Fitts’ Law [31], which posits that the time needed to complete a movement depends on the distance to the target and the target’s size. Children diagnosed with ASD may deliberately reduce their movement speed to enhance accuracy, which can lead to decreased performance on tasks that necessitate both speed and precision. Craig [27] proposed that this trade-off between speed and accuracy might have a more significant impact on pegboard and balance activities. In the current study, this pattern was clearly evident: both ASD groups demonstrated lower manual dexterity scores than TD peers, and the severe ASD group showed the most profound deficits, particularly in balance tasks.

Regarding balance, both ASD groups performed significantly worse than TD children, with the severe ASD group showing the lowest mean scores. Balance impairments in ASD have been consistently reported across diverse samples [32,33,34]. These deficits likely reflect difficulties integrating vestibular, visual, and somatosensory inputs [35,36,37]. Studies comparing eyes-open and eyes-closed balance performance have shown that children with ASD often rely heavily on visual cues for postural control [38], and stability deteriorates markedly when visual input is reduced. Although visual dependence could not be examined in the present study, the significantly lower MABC-2 balance scores in ASD are consistent with a sensory-integration-related mechanism. Importantly, balance was not correlated with IQ, consistent with findings from Luna et al. [39] suggesting that balance deficits are intrinsic to ASD rather than mediated by cognitive ability.

In the current study, joint hypermobility was confined to particular joints, mainly the elbows, instead of indicating a generalized laxity of ligaments. This localized observation implies that biomechanical factors specific to joints, rather than a widespread involvement of connective tissue, could play a role in the motor performance variations seen in children with ASD. This supports findings from studies using Beighton scoring, which report increased hypermobility in ASD [40,41]. Conversely, ankle dorsiflexion was significantly reduced among children with ASD, particularly in the mild-to-moderate subgroup, which aligns with research documenting reduced range of motion at the ankle during gait cycles in ASD [42]. These contrasting findings suggest that joint mobility in ASD may vary depending on anatomical region and functional demand, with some joints demonstrating hypermobility and others restricted movement.

Endurance and physical fitness parameters revealed substantial deficits in both ASD groups. Children with ASD walked significantly shorter distances on the 6 min walk test and performed considerably worse on the standing long jump than their TD peers. These findings mirror prior studies demonstrating lower cardiovascular fitness, reduced physical activity, and diminished muscular power in ASD [43]. LeCheminant [44] similarly found reduced jump distances among individuals with ASD, though that study involved adults rather than children. Beyond motor implications, emerging research such as Yordanova et al. [45] suggests that non-aerobic fitness may be linked to enhanced neural processing (as reflected in P3 amplitude) and improved attentional control, indicating that physical fitness may have cognitive and neurophysiological relevance in ASD.

Muscle strength, as measured by hand grip force, was markedly lower in both ASD groups compared with TD children. This is consistent with earlier work [2,46] indicating that muscle weakness may contribute to broader movement difficulties, impaired balance, and reduced coordination. The strong correlations found between hand grip and MABC-2 performance in aiming and catching, balance, and total score further support the conclusion that muscle strength plays a critical role in overall motor proficiency in ASD. While hand grip is only one measure of strength, reduced values may reflect more widespread neuromuscular involvement.

Flexibility findings were less consistent. Only the right knee angle differed significantly between minimal-to-moderate ASD and TD children, suggesting reduced flexibility in that subgroup, while no significant differences were found for the severe ASD group. This pattern resembles the results of prior research [47], which identified reduced flexibility in ASD but emphasized heterogeneity in presentation and measurement.

The correlations identified among motor performance, IQ, muscle strength, and endurance should be understood within the limitations of the study’s cross-sectional framework. It is not possible to deduce causal relationships, and factors that were not measured, including attention, motivation, task understanding, and behavioral regulation, may have impacted performance, especially in children with diminished intellectual capabilities. Consequently, the results represent functional motor performance in controlled testing environments rather than distinct motor ability. Given that all children with ASD who participated exhibited low intellectual functioning, the motor impairments observed may predominantly represent ASD in conjunction with intellectual disability, rather than autism across the entire cognitive spectrum.

Motor impairments in children diagnosed with ASD have significant implications for their daily functioning and engagement. Challenges in manual dexterity and ball skills can adversely impact handwriting, academic performance, and recreational activities, whereas diminished muscle strength and endurance may restrict autonomy in everyday tasks and involvement in physical activities [29,48]. Additionally, joint-specific hypermobility may further hinder postural stability and elevate the risk of musculoskeletal issues [49]. These observations underscore the necessity of regular motor assessments in children with ASD and emphasize the critical role of integrating focused interventions for motor skills, strength, balance, and endurance within comprehensive rehabilitation programs to improve functional participation and overall quality of life [50,51].

### Limitations

Several limitations must be recognized. Firstly, the sample size was relatively small, predominantly consisted of males, and was confined to a narrow age range, which limits the generalizability of the results. The subgroup with severe ASD was particularly constrained, which diminished the statistical power for comparisons between subgroups and heightened the risk of Type II error. Secondly, all children with ASD in the current sample exhibited low intellectual functioning, which restricts the generalizability to individuals with ASD who do not have co-occurring intellectual disabilities. Thirdly, autism severity was assessed using CARS-2 instead of a gold-standard observational tool like ADOS-2, which limits the accuracy of severity classification and international comparability. Fourthly, the MABC-2 has not been standardized for populations in the Middle East, and interpretations based on percentiles may be affected by cultural and experiential variables. Fifthly, the cross-sectional nature of the study prevents causal inferences, and motor performance outcomes may have been affected by cognitive, attentional, or motivational factors. Lastly, the research was unable to utilize multivariate analyses such as ANCOVA, MANCOVA, or PCA because of a limited sample size, particularly within subgroups of ASD severity, which could result in overfitting and unreliable outcomes. Subsequent studies with larger sample sizes should incorporate multivariate and data-reduction techniques to more effectively identify fundamental motor constructs and account for variables such as age and cognitive ability.

Future investigations ought to utilize larger samples that are both cognitively and geographically diverse, include children with a wide range of cognitive abilities, and employ gold-standard observational diagnostic instruments. Research should also implement longitudinal or interventional methodologies to elucidate causal relationships and developmental pathways of motor impairments in ASD. Furthermore, there is an urgent requirement to create or modify culturally appropriate, reliable motor assessment tools specifically tailored for children with ASD.

## 5. Conclusions

Children with ASD demonstrated significant impairments in gross and fine motor performance, muscle strength, endurance, and selected joint mobility compared with typically developing peers. Associations between motor performance, particularly aiming and catching, and autism severity and intellectual functioning should be interpreted as exploratory, given limitations in severity characterization and sample composition. Nonetheless, the findings underscore the clinical relevance of motor assessment in children with ASD, particularly those with co-occurring intellectual disability, and support the inclusion of targeted motor interventions within comprehensive rehabilitation programs.

## Figures and Tables

**Table 1:** Demographic, anthropometric, and clinical characteristics of all study children.

Characteristics		TD	Mild/Moderate ASD	Severe ASD	*p* Value (Kruskal–Wallis Test)	*p* Value (Mann–Whitney Test)
Number		27	18	8	-	-
Age (Years) (Mean/SD)		7.9 ± 2.2	8.8 ± 1.7	7.5 ± 2.9	0.38	
Sex (No.) (%)	♂	21 (77.8)	16 (88.9)	5 (62.5)	0.31	
	♀	6 (22.2)	2 (11.1)	3 (37.5)		
Handedness (No.) (%)	Rt	24 (88.9)	17 (94.4)	6 (75.0)	0.37	
	Lt	2 (7.4)	0 (0)	1 (12.5)		
	No Preference	1 (3.7)	1 (5.6)	1 (12.5)		
Weight (Kg) (Mean/SD)		25.9 ± 8.3	31.2 ± 10.9	30.1 ± 12.6	0.23	
Height (m) (Mean/SD)		1.3 ± 0.1	1.3 ± 0.1	1.2 ± 0.2	0.53	
BMI [Kg/(m)^2^] (Mean/SD)		15.9 ± 2.7	17.7 ± 3.2	19.1 ± 4.4	0.02 *	
CARS-2 Score (Mean/SD)		Not Documented	31.2 ± 3.6	38.9 ± 2.6	-	<0.001 *
IQ Score (Mean/SD)		Not Documented	64.9 ± 25.5	57.7 ± 16.1	-	0.4

TD: Typically Developed; ASD: Autism Spectrum Disorder; CARS-2: Childhood Autism Rating Scale (2nd Edition); IQ: Intelligence Quotient. * Significant difference (*p* ≤ 0.05).

**Table 2:** Comparing different research groups with respect to gross and fine motor skills (Movement Assessment Battery for Children-2) (Kruskal–Wallis test).

Parameters	Groups (Mean/SD) (Mean Rank)		Chi-Square	Sig.
Manual Dexterity Standard Score	TD (7.1 ± 3.0) (31.5)	MM-ASD(1.9 ± 0.8) (10.3)	29.0	<0.001 *
	TD (7.1 ± 3.0) ((21.7))	S-ASD (2.0 ± 0.9) (5.6)	15.5	<0.001 *
	MM-ASD(1.9 ± 0.8) (13.2)	S-ASD (2.0 ± 0.9) (14.3)	0.1	0.7
Manual Dexterity Percentile	TD (24.7 ± 24.8) (21.7) (31.3)	MM-ASD(0.5 ± 0.4) (10.6)	27.6	<0.001 *
	TD (24.7 ± 24.8) (21.6)	S-ASD (0.5 ± 0.4) (5.9)	14.7	<0.001 *
	MM-ASD (0.5 ± 0.4) (13.2)	S-ASD (0.5 ± 0.4) (14.3)	0.1	0.7
Aiming and Catching Standard Score	TD (10.2 ± 3.0) (30.9)	MM-ASD (3.6 ± 2.6) (11.1)	25.0	<0.001 *
	TD (10.2 ± 3.0) (21.8)	S-ASD (2.4 ± 2.3) (5.3)	16.2	<0.001 *
	MM-ASD (3.6 ± 2.6) (14.8)	S-ASD (2.4 ± 2.3) (10.6)	1.9	0.2
Aiming and Catching Percentile	TD (53.0 ± 30.5) (30.9)	MM-ASD (6.2 ± 11.6) (11.1)	25.0	<0.001 *
	TD (53.0 ± 30.5) (22.0)	S-ASD (0.4 ± 0.2) (4.5)	18.2	<0.001 *
	MM-ASD (6.2 ± 11.6) (15.3)	S-ASD (0.4 ± 0.2) (9.4)	3.8	0.05 *
Balance Standard Score	TD (11.2 ± 2.8) (31.7)	MM-ASD (2.6 ± 2.5) (9.9)	30.6	<0.001 *
	TD (11.2 ± 2.8) (22.0)	S-ASD (1.8 ± 1.5) (4.5)	18.3	<0.001 *
	MM-ASD (2.6 ± 2.5) (14.1)	S-ASD (1.8 ± 1.5) (12.1)	0.6	0.5
Balance Standard Percentile	TD (59.0 ± 30.7) (31.7)	MM-ASD (3.7 ± 8.0) (10.0)	30.0	<0.001 *
	TD (59.0 ± 30.7) (22.0)	S-ASD (0.8 ± 1.7) (4.5)	18.3	<0.001 *
	MM-ASD (3.7 ± 8.0) (14.1)	S-ASD (0.8 ± 1.7) (12.1)	0.6	0.5

TD: Typically Developed; MM-ASD: Mild to Moderate Autism Spectrum Disorder; S-ASD: Severe Autism Spectrum Disorder; * significant difference (*p* ≤ 0.05).

**Table 3:** Comparing different research groups with respect to passive range of motion (Kruskal–Wallis test).

Parameters	Groups (Mean/SD) (Mean Rank)		Chi- Square	Sig.
Passive Range of Motion				
Elbow Flexion	TD (145.6 ± 7.1) (29.5)	MM-ASD (135.7 ± 5.5) (13.3)	17.1	<0.001 *
	TD (145.6 ± 7.1) (20.9)	S-ASD (133.6 ± 8.2) (8.2)	9.8	0.002 *
	MM-ASD (135.7 ± 5.5) (14.0)	S-ASD (133.6 ± 8.2) (12.4)	0.2	0.6
Elbow Hyperextension	TD (2.0 ± 3.5) (20.2)	MM-ASD (5.5 ± 6.2) (27.3)	4.1	0.04 *
	TD (2.0 ± 3.5) (17.0)	S-ASD (4.1 ± 4.6) (21.3)	1.5	0.2
	MM-ASD (5.5 ± 6.2) (13.9)	S-ASD (4.1 ± 4.6) (12.5)	0.2	0.6
Wrist Flexion	TD (97.0 ± 9.5) (22.5)	MM-ASD (96.9 ± 7.6) (23.7)	0.1	0.8
	TD (97.0 ± 9.5) (18.4)	S-ASD (95.8 ± 8.6) (16.8)	0.2	0.7
	MM-ASD (96.9 ± 7.6) (14.3)	S-ASD (95.8 ± 8.6) (11.6)	0.7	0.4
Wrist Extension	TD (97.7 ± 10.4) (26.8)	MM-ASD (87.6 ± 13.2) (17.3)	6.1	0.01 *
	TD (97.7 ± 10.4) (20.3)	S-ASD (87.8 ± 5.3) (10.1)	7.9	0.005 *
	MM-ASD (87.6 ± 13.2) (13.6)	S-ASD (87.8 ± 5.3) (13.4)	0.003	1.0
Finger Flexion	TD (94.9 ± 8.6) (25.4)	MM-ASD (88.9 ± 10.7) (19.3)	3.0	0.08
	TD (94.9 ± 8.6) (18.4)	S-ASD (91.8 ± 3.6) (16.6)	0.3	0.6
	MM-ASD (88.9 ± 10.7) (12.8)	S-ASD (91.8 ± 3.6) (15.1)	0.6	0.4
Finger Extension	TD (80.3 ± 10.1) (20.5)	MM-ASD (84.8 ± 19.2) (26.8)	2.6	0.1
	TD (80.3 ± 10.1) (17.0)	S-ASD (84.8 ± 6.8) (21.5)	1.3	0.3
	MM-ASD (84.8 ± 19.2) (14.1)	S-ASD (84.8 ± 6.8) (12.2)	0.02	0.9
Ankle Dorsiflexion	TD (24.9 ± 7.6) (28.4)	MM-ASD (15.4 ± 9.7) (14.9)	11.8	0.001 *
	TD (24.9 ± 7.6) (18.7)	S-ASD (23.4 ± 9.5) (15.6)	0.6	0.4
	MM-ASD (15.4 ± 9.7) (11.6)	S-ASD (23.4 ± 9.5) (17.9)	4.0	0.05 *
Ankle Plantarflexion	TD (59.8 ± 8.5) (24.1)	MM-ASD (56.4 ± 15.1) (21.3)	0.5	0.5
	TD (59.8 ± 8.5) (18.7)	S-ASD (54.1 ± 15.8) (15.8)	0.5	0.5
	MM-ASD (56.4 ± 15.1) (13.7)	S-ASD (54.1 ± 15.8) (13.1)	0.04	0.8

TD: Typically Developed; MM-ASD: Mild to Moderate Autism Spectrum Disorder; S-ASD: Severe Autism Spectrum Disorder; * significant difference (*p* ≤ 0.05).

**Table 4:** Comparing the different research groups with respect to muscle strength, endurance, and musculoskeletal flexibility (Kruskal–Wallis test).

Parameters	Groups (Mean/SD) (Mean Rank)		Chi- Square	Sig.
Muscle Strength				
Hand Grip (Right)	TD (11.5 ± 4.4) (30.4)	MM-ASD (4.5 ± 2.9) (11.9)	21.5	<0.001 *
	TD (11.5 ± 4.4) (21.6)	S-ASD (3.6 ± 3.0) (5.9)	14.5	<0.001 *
	MM-ASD (4.5 ± 2.9) (14.3)	S-ASD (3.6 ± 3.0) (11.8)	0.6	0.4
Hand Grip (Left)	TD (10.7 ± 4.7) (29.7)	MM-ASD (3.9 ± 3.5) (12.9)	17.8	<0.001 *
	TD (10.7 ± 4.7) (21.4)	S-ASD (3.4 ± 2.9) (6.5)	13.1	<0.001 *
	MM-ASD (3.9 ± 3.5) (13.4)	S-ASD (3.4 ± 2.9) (13.8)	0.01	0.9
Average Hand Grip	TD (11.1 ± 4.5) (30.2)	MM-ASD (4.2 ± 3.1) (12.3)	20.0	<0.001 *
	TD (11.1 ± 4.5) (21.5)	S-ASD (3.5 ± 2.8) (6.3)	13.6	<0.001 *
	MM-ASD (4.2 ± 3.1) (13.9)	S-ASD (3.5 ± 2.8) (12.6)	0.2	0.7
Endurance				
6-Minutes Walking Distance	TD (500.6 ± 109.5) (30.5)	MM-ASD (353.3 ± 48.1) (11.8)	21.9	<0.001 *
	TD (500.6 ± 109.5) (21.4)	S-ASD (309.4 ± 141.4) (6.4)	13.3	<0.001 *
	MM-ASD (353.3 ± 48.1) (13.6)	S-ASD (309.4 ± 141.4) (13.2)	0.02	0.9
Standing Long-Jump Distance	TD (136.3 ± 46.3) (31.6)	MM-ASD (21.8 ± 21.6) (10.2)	28.7	<0.001 *
	TD (136.3 ± 46.3) (20.9)	S-ASD (11.9 ± 20.3) (4.3)	15.6	<0.001 *
	MM-ASD (21.8 ± 21.6) (14.1)	S-ASD (11.9 ± 20.3) (10.1)	1.7	0.2
Musculoskeletal Flexibility				
Knee Angle (Right)	TD (2.8 ± 5.3) (22.2)	MM-ASD (12.2 ± 17.4) (27.2)	4.1	0.04 *
	TD (2.8 ± 5.3) (16.1)	S-ASD (8.3 ± 9.8) (21.3)	2.2	0.1
	MM-ASD (12.2 ± 17.4) (12.6)	S-ASD (8.3 ± 9.8) (12.1)	0.03	0.9
Knee Angle (Left)	TD (3.1 ± 5.6) (20.4)	MM-ASD (13.6 ± 19.2) (26.9)	3.6	0.06
	TD (3.1 ± 5.6) (16.1)	S-ASD (8.3 ± 9.8) (20.9)	1.7	0.2
	MM-ASD (13.6 ± 19.2) (12.7)	S-ASD (8.3 ± 9.8) (12.0)	0.05	0.8
Average Knee Angle	TD (3.0 ± 5.2) (20.4)	MM-ASD (12.9 ± 18.2) (27.0)	3.6	0.06
	TD (3.0 ± 5.2) (16.1)	S-ASD (8.3 ± 9.8) (21.2)	1.9	0.2
	MM-ASD (12.9 ± 18.2) (12.7)	S-ASD (8.3 ± 9.8) (12.0)	0.05	0.8

TD: Typically Developed; MM-ASD: Mild to Moderate Autism Spectrum Disorder; S-ASD: Severe Autism Spectrum Disorder; * significant difference (*p* ≤ 0.05).

**Table 5:** Association between Movement Assessment Battery for Children-2, motor performance, clinical, and intellectual characteristics in children with autism.

Independent Parameters	Spearman Correlation							
	Movement Assessment Battery for Children-2 (MABC-2)							
	MDSS		ACSS		BSS		TSS	
	r	*p*	r	*p*	r	*p*	r	*p*
CARS-2	−0.16	0.43	−0.38	0.05 *	−0.12	0.57	−0.30	0.14
IQ	0.17	0.49	0.51	0.03 *	0.002	1.00	0.15	0.55
Hand Grip Right	0.23	0.26	0.59	0.001 **	0.36	0.07	0.39	0.05 *
Hand Grip Left	0.01	0.95	0.48	0.01 *	0.49	0.01 *	0.44	0.02 *
Average Hand Grip	0.14	0.49	0.55	0.003 **	0.44	0.02 *	0.43	0.03 *
6-Minute Walking Distance	0.22	0.29	0.58	0.002 **	0.31	0.12	0.42	0.03 *
Standing Long-Jump Distance	0.12	0.57	0.44	0.03 *	0.32	0.12	0.26	0.20

CARS-2: Childhood Autism Rating Scale (2nd Edition); IQ: Intelligence Quotient; MDSS: Manual Dexterity Standard Score; ACSS: Aiming and Catching Standard Score; BSS: Balance Standard Score; TSS: Total Standard Score. r: Spearman correlation coefficient. * Correlation is significant at the 0.05 level. ** Correlation is significant at the 0.01 level.

## Data Availability

The data supporting the findings and analyzed during this study are available from the corresponding author upon reasonable request.

## Abbreviations

The following abbreviations are used in this manuscript:

ASD	Autism Spectrum Disorder
ADHD	Attention Deficit Hyperactivity Disorder
TD	Typically Developing
CARS-2	Childhood Autism Rating Scale—Second Edition
IQ	Intelligence Quotient
MABC-2	Movement Assessment Battery for Children—Second Edition
SB5	Stanford–Binet Intelligence Scale, Fifth Edition
VIQ	Verbal IQ
PIQ	Performance IQ
ROM	Range of Motion
M6MWT	Modified 6-Minute Walk Test
SLJ	Standing Long Jump
FMS	Fundamental Movement Skill
IRB	Institutional Review Board
